# Supplementary figures and images for: Chlamydia Psittaci ST24: Clonal Strains of One Health Importance Dominate in Australian Horse, Bird and Human Infections
Source: Pathogens. 2021 Aug 11;10(8):1015. doi: 10.3390/pathogens10081015 (PMC8401489; doi:10.3390/pathogens10081015)

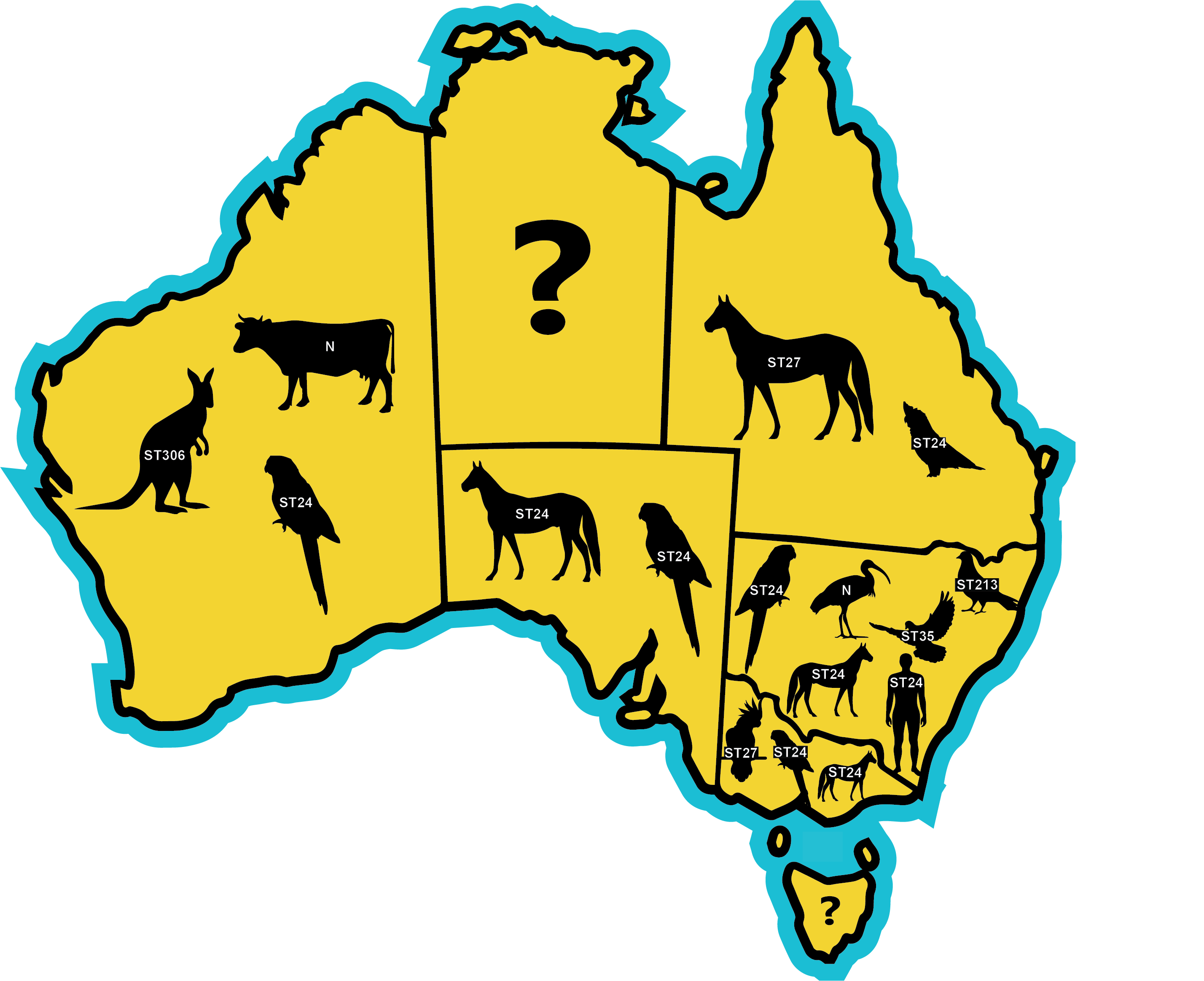

Supplement: Supplementary file 1 [file pathogens-10-01015-s001.zip › Supplementary Files/FigureS2.png]
